# Randomized controlled trial of transcutaneous electrical nerve stimulation for pain relief during transvaginal oocyte retrieval using conscious sedation: study protocol for a randomized controlled trial

**DOI:** 10.1186/s13063-019-3227-5

**Published:** 2019-04-11

**Authors:** Queenie Ho Yan Wong, Man Wa Lui, Sofie Shuk Fei Yung, Jennifer Ka Yee Ko, Raymond Hang Wun Li, Ernest Hung Yu Ng

**Affiliations:** 1 Department of Obstetrics and Gynaecology, The University of Hong Kong Queen Mary Hospital, 6/F, Professorial Block, Hong Kong, People’s Republic of China; 2 Department of Obstetrics and Gynaecology, Princess Margaret Hospital, Hong Kong, People’s Republic of China

**Keywords:** Transcutaneous electrical nerve stimulation, Pain relief, Transvaginal oocyte retrieval, Conscious sedation

## Abstract

**Background:**

Transvaginal oocytes retrieval is an essential step in in-vitro fertilization treatment. There are different pain relief methods, but none has been shown to be superior than the others. Transcutaneous electrical nerve stimulation (TENS) is a non-pharmacological and non-invasive pain relief method. This study aims to compare the pain levels experienced by the women using the conscious sedation and those who had TENS in addition to conscious sedation.

**Methods and analysis:**

This is a double-blinded randomized trial that will be carried out in a university-assisted conception unit. Women who will undergo oocyte retrieval under conscious sedation will be recruited. After randomization, women will be allocated to either the active TENS group or placebo TENS group (the TENS machine will not emit active impulse), in addition to the paracervical block and conscious sedation. The primary outcome is pain levels of women during the retrieval assessed by the visual analog scale. Secondary outcomes include satisfaction of women and postoperative side effects.

**Discussion:**

TENS is an effective non-pharmacological and non-invasive method for pain relief in a number of clinical conditions. Both women and assisted conception unit can benefit if the addition of non-invasive, simple, and low-cost TENS application is proven to be superior than using conscious sedation and paracervical block alone.

**Trial registration:**

ClinicalTrials.gov, NCT03472430. Registered on 3 May 2018.

**Electronic supplementary material:**

The online version of this article (10.1186/s13063-019-3227-5) contains supplementary material, which is available to authorized users.

## Background

Oocyte retrieval under transvaginal ultrasound guidance is an essential part of in-vitro fertilization (IVF). It involves passing an aspiration needle into the pelvic cavity through the vaginal mucosa and puncturing the ovarian cortex to reach the ovarian follicles. Despite being less invasive when compared with retrieval through laparoscopy in the old days, it is still a painful procedure. Different methods of pain relief have been used. The most commonly used modalities are conscious sedation with analgesia with or without paracervical block [1–3], patient-controlled conscious sedation, and analgesia [4], spinal anesthesia, and general anesthesia.

The best type of analgesia has not been established. A Cochrane review in 2013 [5] on pain relief for women undergoing oocyte retrieval for assisted reproduction concluded that the current evidence from 21 randomized controlled trials (RCTs) did not support one particular method over another. The concurrent use of more than one method of sedation and pain relief resulted in better pain relief than a single modality.

The pain relief method used should be safe, effective, and with minimal side effects. Transcutaneous electrical nerve stimulation is a non-pharmacological and non-invasive pain relief for nociceptive, neuropathic, and musculoskeletal pain involving delivering pulsed electric current across skin [6]. It reduces labor pain and postpones the use of pharmacological analgesics in laboring women [7]. It has also been reported as reducing pain and increasing patients’ satisfaction when used in office hysteroscopy without sedation [8]. It has not been studied as a pain relief method for oocyte retrieval in assisted reproduction.

## Objectives and hypothesis

This trial aims to compare the pain levels experienced by the women using the conscious sedation and those who had TENS in addition to conscious sedation. The hypothesis is that there will be less pain in women who receive both conscious sedation and TENS during the retrieval procedure.

## Methods

### Trial design

**Fig. 1:**
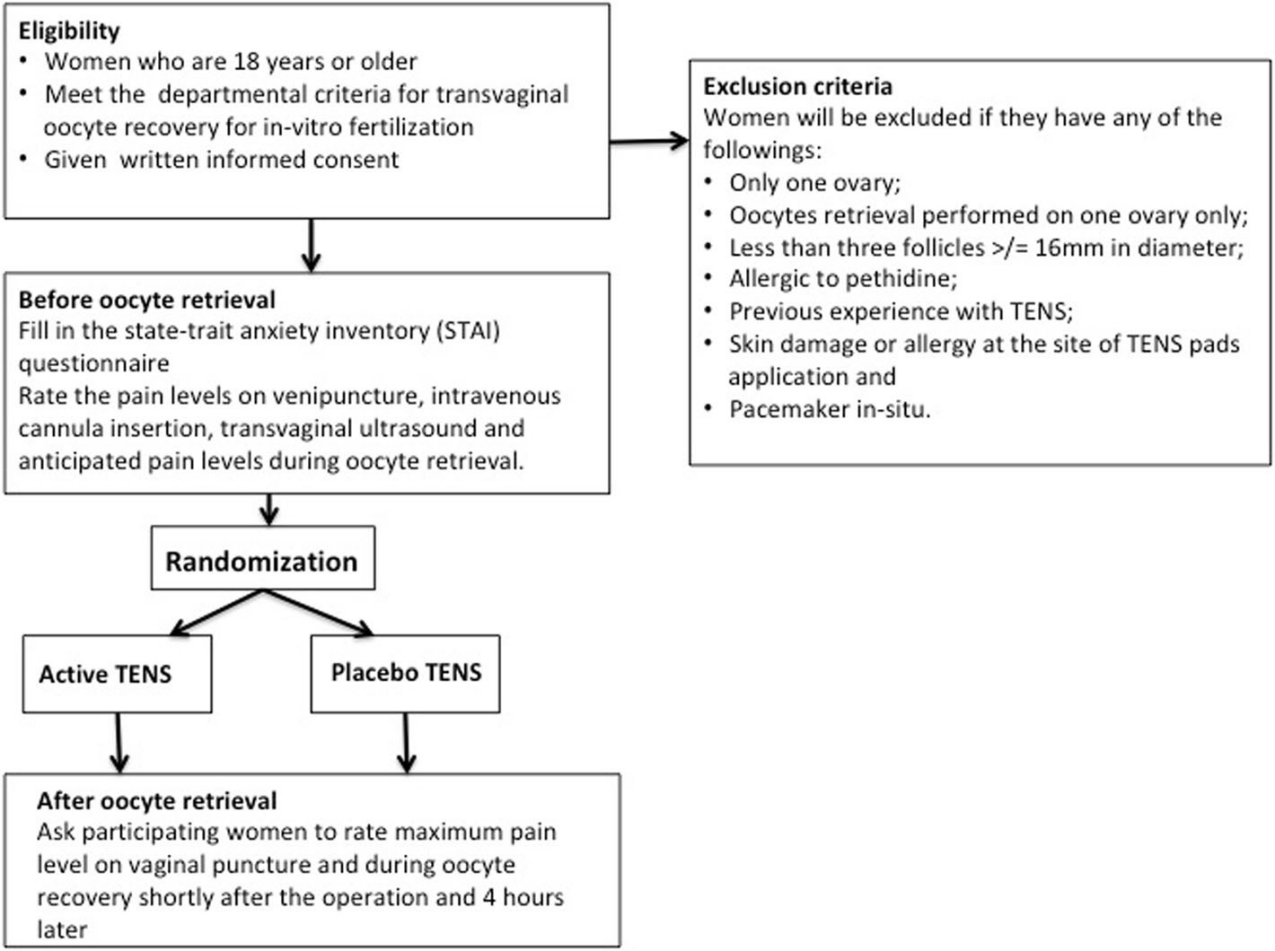
Trial design. The flow chart summarises the design of the trial

**Fig. 2:**
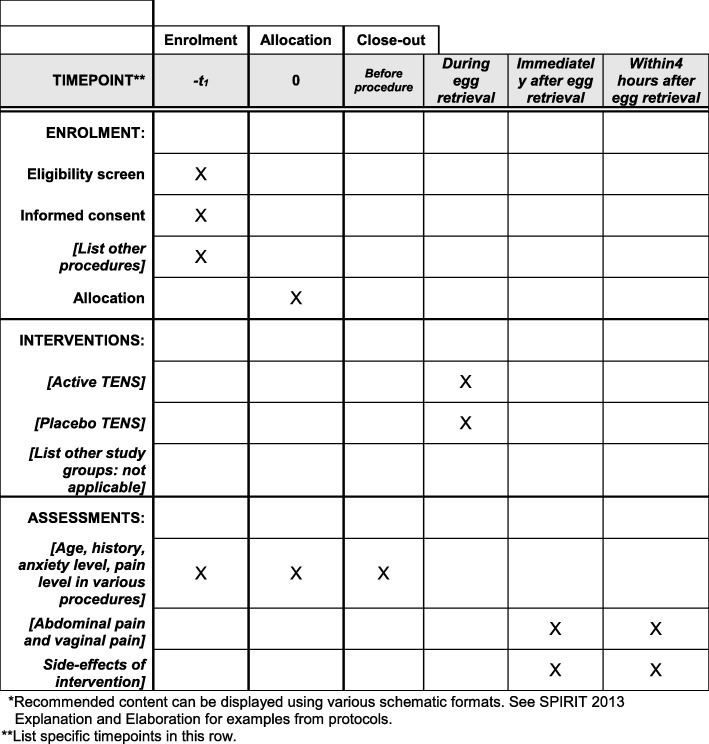
The schedule of enrolment, interventions, and assessments

This is a double-blinded RCT that will be carried out in the assisted reproductive unit in Queen Mary Hospital in Hong Kong. Queen Mary Hospital is affiliated to the University of Hong Kong. The study has been approved by the Institutional Review Broad of the University of Hong Kong/Hospital Authority Hong Kong West Cluster and has been registered under Clinical Trials Registry (trial number NCT03472430). Written consent will be obtained from women at the time of recruitment by medical staff. The clinical trial protocol follows the Additional file 1. Figures 1 and 2 summarize the trial design with the details of the trial as described below.

### Selection and withdrawal of participants

Women undergoing ovarian stimulation for IVF in the unit will be informed of the study. Women aged ≥ 18 years, who have met the criteria for transvaginal oocyte recovery for IVF according to departmental protocol (a dominant follicle of at least 18 mm and three more follicles of at least 16 mm in diameter) will be recruited. Women will be excluded if they have any of the followings: (1) only one ovary; (2) oocyte retrieval performed on one ovary only; (3) less than three follicles ≥ 16 mm in diameter; (4) allergy to pethidine; (5) previous experience with TENS; (6) skin damage or allergy at the site of TENS pads application; and (7) pacemaker in situ.

The participation in the study is entirely voluntary. Consented women can withdraw their consent at any time and they will receive standard medical care according to the departmental protocol.

### Randomization and masking

Randomization is carried out in blocks by a computer-generated randomization list generated by a research nurse not involved in the women’s clinical management. Women will be randomized into one of the two groups on the day of the oocyte retrieval: (1) Treatment group: active TENS; and (2) Placebo group: placebo TENS.

The clinicians, nurses involved in the clinical management of women, and the women will all be blinded to the treatment allocation. Checking of blinding for the women, surgeons, and scrub nurses will be assessed.

### Procedure

Written consent will be obtained on the day of ovulation trigger. Consent to participate in the study will be confirmed again on the day of the retrieval. Randomization will be done on the day of oocyte retrieval as described above, according to a computer-generated randomization code in sealed envelopes prepared by a dedicated research nurse who is not involved in usual clinical care.

Women will then complete the state-trait anxiety inventory (STAI) questionnaire [9] before oocyte retrieval to assess their trait and state anxiety. Before the retrieval procedure, two sets of self-adhesive electrodes will be placed on the women back on each side of the spine at T10 to S4 level.

Instructions on how to titrate the TENS amplitude to the desired level that gives a strong non-painful electrical paresthesia will be given to the women by another research nurse who is unaware of the group allocation. TENS therapy will start 5 min before the procedure and stop 5 min after the removal of aspiration needle. The TENS machine used in the study is Endomed 482 that delivers biphasic pulsed currents using pulse duration of 400 μs and pulse frequencies between 80 and 100 pulses s^−^. The women in the treatment group will be given a TENS machine with electrodes emitting impulses while those in the placebo group will be given a TENS machine with electrodes that is not emitting any impulses. A nurse responsible for the study who is unaware of the randomization is responsible for ensuring administration of intervention before oocyte retrieval and withdrawal of intervention after the procedure.

The woman will lie on the operative bed in a lithotomy position. In total, 25 mg pethidine and 5 mg diazepam will be given intravenously. The blood pressure and pulse of the woman are checked after the drug administration and the oxygen saturation is monitored continuously throughout the procedure. A total of 10 mL of 1% lignocaine is injected to the paracervical region by the operating surgeon with a 21-gauge needle after cleansing the vagina and cervix with chlorhexidine. Under transvaginal ultrasound guidance, a 16-gauge ovum aspiration needle is introduced. Aspiration of follicles is performed with a suction pressure of 100 mmHg.

### Assessment of pain and sedation levels

Before oocyte retrieval, a nurse who will not be involved in the operation and is blinded to the intervention will ask the women to rate the pain levels according to a 100-mm linear visual analog scale on venipuncture, intravenous cannula insertion, transvaginal ultrasound, and anticipated pain levels during oocyte retrieval. The maximum pain level on vaginal puncture and during oocyte recovery will be rated by the women within 5 min after the operation. At 4 h after the operation before discharge, the women will be asked to rate the maximum vaginal and abdominal pain as well as satisfaction level towards pain relief (totally unsatisfactory, not very satisfactory, fair, satisfactory, or excellent). The nurse will document if there are any side effects (nausea, vomiting, dizziness, drowsiness, skin allergy at site of TENS pad application, discomfort due to electrical stimulation) from pain relief.

The surgeon will score the sedation level of the women according to the scale described by Ramsay et al. [10], at the end of the operation: level 1 = patient anxious and agitated or restless or both; level 2 = patient cooperative, orientated, and tranquil; level 3 = patient responsive to commands only; level 4 = asleep and a brisk response to loud auditory stimulus; level 5 = asleep and a sluggish response to loud auditory stimulus; and level 6 = asleep and no response to loud auditory stimulus.

### Clinical data

Data on the dosage of drug use for conscious sedation, time taken for oocyte retrieval, number of follicles aspirated, and number of oocytes obtained will be documented. Clinical information on cause of infertility, previous obstetrics history, smoking status, body mass index, cycle stimulation, number of embryos transferred, and outcomes of the IVF cycle will be recorded.

## Statistics

The primary outcome will be the pain level during oocyte retrieval experienced by women. Secondary outcomes include postoperative side effects and satisfaction of women.

According to our previous study [3], the pain level of the retrieval using conscious sedation alone was 23.0 ± 21.0 (mean ± SD) on a 100-point visual analogue scale. Effect size is estimated based on recommendations for a minimum clinically relevant change of 10 mm on a 0–100 mm VAS for pain. Assuming the pain level is reduced from 23.0 to 13.0 on a 100-point visual analogue scale following TENS, the sample size required would be 70 in each treatment arm to give a test of significance of 0.05 and a power of 0.8 (Sigmastat, Jandel Scientific, USA). A total of 160 participants or 80 participants in each treatment group are needed if we anticipated a dropout rate of 10%.

### Statistical analysis

Statistical analysis will be performed with intention-to-treat and per-protocol analyses. Demographic features, ovarian response parameters, and operative time between the two groups will be compared. The Mann–Whitney test will be used to compare differences in VAS between the two groups for primary outcome, which is usually skewed. Chi-square test will be used to analyze the presence of side effects. Level of sedation and women’s satisfaction will be compared using the χ^2^ test. All statistical analyses will be performed using the Statistical Program for Social Sciences, SPSS software. Statistical significance will be taken as *P* < 0.05.

## Discussion

Many oocyte retrievals are performed in assisted conception units and there are different pain relief methods during the retrieval procedure. However, there is no evidence that any pain relief method is superior than the others.

TENS is an effective non-pharmacological and non-invasive method for pain relief in a number of clinical conditions. Women can benefit if the addition of non-invasive, simple, and low-cost TENS application is proven to be superior than using conscious sedation and paracervical block alone. There are risks to women with deep sedation, epidural anesthetics, and general anesthetics despite them being effective ways for pain relief. These methods also require the service of anesthetists. Therefore, the use of TENS can have the potential to improve the safety of women and to benefit a service unit from a cost-effectiveness perspective.

### Trial status

The trial started recruitment in May 2018 and had recruited 45 patients.

## Additional file

Additional file 1: SPIRIT 2013. (DOC 121 kb)
